# RETRACTED: Yue et al. A Ferulic Acid Derivative FXS-3 Inhibits Proliferation and Metastasis of Human Lung Cancer A549 Cells via Positive JNK Signaling Pathway and Negative ERK/p38, AKT/mTOR and MEK/ERK Signaling Pathways. *Molecules* 2019, *24*, 2165

**DOI:** 10.3390/molecules30102139

**Published:** 2025-05-13

**Authors:** Shi-Jun Yue, Peng-Xuan Zhang, Yue Zhu, Nian-Guang Li, Yan-Yan Chen, Jia-Jia Li, Sai Zhang, Ru-Yi Jin, Hao Yan, Xu-Qin Shi, Yu-Ping Tang, Jin-Ao Duan

**Affiliations:** 1 Key Laboratory of Shaanxi Administration of Traditional Chinese Medicine for TCM Compatibility, and State Key Laboratory of Research and Development of Characteristic Qin Medicine Resources (Cultivation), and Shaanxi Key Laboratory of Chinese Medicine Fundamentals and New Drugs Research, and Shaanxi Collaborative Innovation Center of Chinese Medicinal Resources Industrialization, Shaanxi University of Chinese Medicine, Xi’an 712046, Shaanxi Province, China; shijun_yue@163.com (S.-J.Y.); oucysj@126.com (P.-X.Z.); chenyanyan59@163.com (Y.-Y.C.); ljjzhw2007@163.com (J.-J.L.); zs18740484737@163.com (S.Z.); jinruyi335588@163.com (R.-Y.J.); yanhaohainan@126.com (H.Y.); 2 Jiangsu Collaborative Innovation Center of Chinese Medicinal Resources Industrialization, and Jiangsu Key Laboratory for High Technology Research of TCM Formulae, and National and Local Collaborative Engineering Center of Chinese Medicinal Resources Industrialization and Formulae Innovative Medicine, Nanjing University of Chinese Medicine, Nanjing 210023, Jiangsu Province, China; zhuyue@njucm.edu.cn (Y.Z.); linianguang@njucm.edu.cn (N.-G.L.); shixuqin@126.com (X.-Q.S.); dja@njucm.edu.cn (J.-A.D.)

The journal retracts the article titled “A Ferulic Acid Derivative FXS-3 Inhibits Proliferation and Metastasis of Human Lung Cancer A549 Cells via Positive JNK Signaling Pathway and Negative ERK/p38, AKT/mTOR and MEK/ERK Signaling Pathways” [1], as cited above.

Following publication, concerns were brought to the attention of the Editorial Office regarding inappropriate image modification and duplication contained within this article [1].

Adhering to our standard procedure, the Editorial Office and Editorial Board conducted an investigation that confirmed unusual image anomalies and duplication of sub-images in Figure 5B [1] and duplication of sub-images in Figure 5B,C [1], presenting different experimental conditions. While the authors collaborated with the Editorial Office during the investigation, they were unable to satisfactorily explain the overlapping visual features, and the Editorial Board could not confirm the validity of the images from the raw material provided. As a result, the Editorial Board has lost confidence in the validity of the findings and decided to retract this publication [1], as per MDPI’s retraction policy (https://www.mdpi.com/ethics#_bookmark30).

This retraction was approved by the Editor-in-Chief of the *Molecules* journal.

The authors agreed to this retraction.
